# Detecting protein variants by mass spectrometry: a comprehensive study in cancer cell-lines

**DOI:** 10.1186/s13073-017-0454-9

**Published:** 2017-07-18

**Authors:** Javier A. Alfaro, Alexandr Ignatchenko, Vladimir Ignatchenko, Ankit Sinha, Paul C. Boutros, Thomas Kislinger

**Affiliations:** 1 Informatics Program, Ontario Institute for Cancer Research, Toronto, Ontario Canada; 2 Department of Medical Biophysics, University of Toronto, Toronto, Ontario Canada; 3 Princess Margaret Cancer Centre, University Health Network, Toronto, Ontario Canada; 4 Department of Pharmacology & Toxicology, University of Toronto, Toronto, Ontario Canada

**Keywords:** Proteogenomics, Proteoforms, Protein mutant detection, Integrative –omics, Protein search databases, Personalized proteomics, Proteomics, Mass-spectrometry-based mutant detection

## Abstract

**Background:**

Onco-proteogenomics aims to understand how changes in a cancer’s genome influences its proteome. One challenge in integrating these molecular data is the identification of aberrant protein products from mass-spectrometry (MS) datasets, as traditional proteomic analyses only identify proteins from a reference sequence database.

**Methods:**

We established proteomic workflows to detect peptide variants within MS datasets. We used a combination of publicly available population variants (dbSNP and UniProt) and somatic variations in cancer (COSMIC) along with sample-specific genomic and transcriptomic data to examine proteome variation within and across 59 cancer cell-lines.

**Results:**

We developed a set of recommendations for the detection of variants using three search algorithms, a split target-decoy approach for FDR estimation, and multiple post-search filters. We examined 7.3 million unique variant tryptic peptides not found within any reference proteome and identified 4771 mutations corresponding to somatic and germline deviations from reference proteomes in 2200 genes among the NCI60 cell-line proteomes.

**Conclusions:**

We discuss in detail the technical and computational challenges in identifying variant peptides by MS and show that uncovering these variants allows the identification of druggable mutations within important cancer genes.

**Electronic supplementary material:**

The online version of this article (doi:10.1186/s13073-017-0454-9) contains supplementary material, which is available to authorized users.

## Background

A global effort is underway by cancer researchers to annotate biobanks with molecular data captured across the genome, transcriptome, and proteome. While the genomics and transcriptomics communities have established pipelines for the identification of disease variants, it remains difficult to elucidate the consequences of these variations on the proteome. There is a need for better methodologies to characterize all protein variants, formally defined as proteoforms [1], from global proteomics datasets. This includes germline, somatic, and post-translational modifications (PTMs), including all possible combinations, for any given protein. However, the identification of PTMs and coding consequences of genomic variations are conceptually different, since genomic and transcriptomic studies can provide orthogonal evidence for the existence of such a variant.

A fundamental task in mass-spectrometry (MS)-based proteomics is the assignment of collected spectra to the amino-acid sequences that gave rise to them. Proteins are digested using enzymes with known cleavage sites to produce peptides, which are then analyzed by MS. These datasets consist of two types of measurements: (1) MS^1^ spectra survey a set of peptides present in the mass-spectrometer at a given moment; and (2) MS^2^ spectra originate from an attempt to isolate and fragment a single peptide ion species identified in the MS^1^. Peptide spectrum matches (PSMs) are assigned using search algorithms [2–4] that match MS^2^ spectra to peptides originating from a database of reference protein sequences. Typically, a target-decoy approach [5, 6] is used to estimate the false discovery rate (FDR), allowing users to produce a final list of identifications at a selected confidence level.

Generally, the proteomics community has aimed to simplify these search databases by using canonical sequence representatives of each protein in the human proteome. The rationale has been to reduce the peptide search space in order to avoid spurious matching and extensive peptide inference (i.e. peptides matching to more than one database entry) [7]. Difficulties in assigning spectra originate from a variety of factors including low abundance, non-peptide molecules, modified peptides, or mixtures of co-fragmenting peptides. The larger the search database the higher the likelihood of a spurious match [8].

However, one limitation of using reference sequence databases is that it is unclear how the cancer genome, with all its mutations, structural variations, and epigenetic modifications, manifests in a cancer proteome. Onco-proteogenomics expands search databases with protein sequences not found in reference human proteomes, such as germline variations, variants commonly found in cohorts of tumor samples, or sample-specific variants identified in genomic or transcriptomic analyses. Global MS-based proteomic strategies, in combination with genomics and transcriptomics, could resolve this gap in knowledge [9–18] with the goal of improving the characterization of the variant peptides (i.e. peptidoforms) present in the sample.

Two types of databases are commonly used to incorporate protein variants into MS searches: community-based databases include variations previously observed while sample-specific databases include variants identified by DNA sequencing (DNA-seq) or RNA sequencing (RNA-seq) of that sample [19]. Each approach has advantages and disadvantages. Large databases like dbSNP [20], COSMIC [21], and UniProt [22] contain millions of protein variants, which can increase the likelihood of spurious database hits due to the increased database size. By contrast, sample-specific databases may be smaller, but are prone to false negatives resulting from variants missed in DNA-seq or RNA-seq for experimental or computational reasons [23, 24]. Intratumoral heterogeneity adds yet another potential source of missed variant protein detection [25].

State-of-the-art MS is now reaching the resolution and sensitivity to interrogate protein variations [26]. In parallel, the computational developments needed to combine proteomics with DNA-seq and RNA-seq in cancer samples are already underway [12, 19, 27–35]. Here, using proteomic, transcriptomic, and genomic characterization of the NCI60 cell-lines, we systematically investigate how the choice of proteogenomic databases affects PSM assignment. We present a strategy for onco-proteogenomics to assess the scope of variant peptides identified and their potential impact to cancer biology.

## Methods

We conducted our study within the NCI60 cell-line panel with extensive genomic [36], transcriptomic [37], and proteomic [38] data available. The proteomics data consist of both a “deep” proteome derived from extensive fractionation of cell lysate by electrophoresis into 24 gel pieces (nine cell-lines) and a “shallow” proteome, which was generated using 12 gel pieces (59 cell-lines).

### Variant peptide database construction

The first step in variant protein identification was the generation of protein sequence databases containing the modified amino-acid sequences (Additional file 1: Figure S1a). Briefly, protein-level outputs from variant effect predictor [39] were parsed to proteins containing single amino-acid variants, insertions, deletions, frameshifts, stop-loss mutations, and fusions. Variant peptides were filtered against a canonical human proteome from UniProt (20,187 non-redundant proteins) to remove peptides that also mapped to this reference database. Variant sequences longer than six amino acids and containing up to two missed tryptic cleavages on either side of the mutated site were produced and added to the FASTA file.

We explored variant-peptide detection with regards to proteogenomic database size and content. Variant proteins were obtained from five different sources: dbSNP [20]; COSMIC [21]; UniProt [22]; exome-seq [36]; and RNA-seq [37]. Augmented search databases were created in 23 different ways derived from combinations and subsets of these databases (Additional file 1: Figure S1b; Additional file 2). We defined community-based databases to include dbSNP, COSMIC, and variants annotated in UniProt. Four sub-databases of COSMIC and dbSNP were made to include single nucleotide variants, indels, variants affecting genes in the COSMIC cancer gene census and frameshifts, or stop losses or fusions. For sample-specific database searches, all 59 NCI60 cell-lines containing exome-seq data and 41 cell-lines containing RNA-seq data were used. Three further databases restricted to subsets of variants were generated for a total of four sample-specific databases per cell-line and per analyte type. We combined sample-specific and community-based databases in two different ways: we used a sample specific approach and a general approach where all RNA-seq and exome-sequencing (exome-seq) datasets were merged. In total, the RNA-seq cell-line data characterized 675 cell-lines, which were also included separately in their own database, as was all the exome-seq data. A total of 473 different database combinations (Additional file 3; Additional file 1: Figure S1b) were explored across all available cancer cell-lines.

### COSMIC, dbSNP, and exome-seq databases

COSMIC (v70), dbSNP (v141), and processed exome-seq [38] datasets were downloaded in VCF format and parsed using Variant Effect Predictor [39] (VEP.v.77) from Ensembl tools release (v77) using the GRCh37 genome reference model. VEP output files were further parsed to introduce mutations by retrieving the described reference sequences from the Ensembl proteome (GRCh37.75) and applying described substitutions, insertions, and deletions using a series of Bioconductor R scripts (R:v3.1.0; stringer:v0.6.2; cleaver:v1.2.0; Biostrings:2.32.1; Rsamtools:v1.16.1; GenomicFeatures:v1.16.2). Peptides were generated from these mutated sequences allowing for up to two missed cleavage sites. Duplicate peptides were collapsed and headers identifying each mutation merged together.

### COSMIC fusions

Gene fusions were obtained from those manually curated from peer-reviewed publications by COSMIC curators [21]. Fusions lacking inversions were parsed from COSMIC HGVS format by extracting appropriate transcripts (from the GRCh37.75 Ensembl genome model) and merging the corresponding sequences. Tryptic peptides spanning a three-frame translation over the fusion were added to the FASTA database for proteogenomic searching. Note: inversions and more complex fusions were not included in our analysis.

### RNA-seq

RNA-seq datasets were obtained from the authors [37] as tab-delimited files with each mutation fully characterized within a RefSeq protein. Each line in the file was parsed using in-house R scripts to generate mutated protein sequences. Tryptic peptides with up to two missed cleavages were generated overlapping the mutation site. RNA-seq in-frame fusions were made by merging nucleotide sequences for the 5’ and 3’ regions of the fusion. All tryptic peptides spanning the fusion crossover were added to the database.

### UniProt variants

The UniProt database was downloaded in XML format (December 2015) and variants described therein were parsed and corresponding UniProt reference sequences modified.

### Variant peptide detection

Using these databases, variant peptides were identified from the NCI60 cell-lines using a proteogenomic pipeline implementing a split target-decoy approach [15], three search algorithms [2–4], and several additional filters (Additional file 1: Figure S2a, b; Additional file 4; Additional file 5). These filters (1) removed sequences mapping to the human proteome as described above (RefSeq, Ensembl, and UniProt), (2) removed peptide-spectrum-matches that could also be based on chemical or PTMs of reference peptide sequences, and (3) removed protein variants with no alternative evidence for their expression.

### Target decoy database construction

For each FASTA file above, sequences were combined with reviewed canonical Swiss-Prot (v.2014.12.09) protein sequences and each combined sequence was reversed. These original and reversed sequences were merged together to create proteogenomics FASTA databases used for peptide-spectrum match assignment.

### Target decoy database searching

MS RAW files were converted to mzXML format using ReAdW (http://tools.proteomecenter.org/software.php) and searched against the proteogenomics FASTA databases with X!Tandem [2] (v.13.09.01.1), Comet [3] (v.2014.02 r2), and MS-GF+ [4] (v.0.9949). The following search parameters were used for all searches: carbamidomethylation of cysteine as a static modification, oxidation of methionine as a dynamic modification, a ±10 ppm precursor mass tolerance, a ±0.4 Dalton fragment mass tolerance for CID, and ±10.0 ppm fragment mass tolerance for HCD. All searches were performed on a 22-node cluster with 12 cores and 64 GB RAM on each node. Output files were converted into tab-delimited files that standardized outputs from all search algorithms (Additional files 6, 7, 8 and 9). The search results were then subjected to a series filtration steps, described next.

#### Spectral-level FDR cutoff

We calculated spectral-level FDR cutoffs using a split target-decoy approach as initially proposed in [15]. FDR was calculated separately for variant peptides and UniProt PSMs using decoys generated from each database, respectively, although MS data were searched against one merged FASTA file. In each case, PSMs with different mass-to-charge ratios were treated separately. PSMs with less than 1% spectral FDR were retained for subsequent analyses.

### Filtering of resulting peptide lists

Applying a stringent spectral-level FDR filter does not guarantee that every PSM represents a correct identification, especially when single peptide identifications are involved, as is the case in proteogenomics. A number of scenarios could result in false-positive identifications. The detected peptide may be an adjacent tryptic peptide not overlapping the variant, which can arise from FASTA sequences containing missed tryptic cleavage sites included within the database. A variant peptide could be correctly assigned to the spectrum, but inadvertently also match to or be isobaric with a sequence of a different reference protein. A peptide could be erroneously matched to a spectrum, because the mass shift caused by a substitution happens to coincide with the mass shift associated with a PTM on the same or possibly different peptide. Finally, when searching large databases, false-positive rates can be harder to control because there is a higher probability of matching a high scoring peptide from among the larger number of sequences available. We developed a series of post-search filters to mitigate these potential caveats. In the future, these approaches could be further refined, using either synthetic spectral libraries or more sophisticated statistical approaches.

#### Filtration against reference proteomes

A filter was required to deal with scenarios where detected peptides inadvertently matched or could not be distinguished from peptides in the reference proteome. Detected peptides were matched against reference proteomes including that of Ensembl (GRCh37.75), RefSeq (release 68), and UniProtKB/Swiss-Prot sequences. Isobaric leucine and isoleucine residues, which cannot be distinguished, were considered identical during this filtration process.

#### Chemical modification filter

Mass shifts in MS^2^ spectra could also be attributed to PTMs (chemical or enzymatic) within some reference peptide sequence. To deal with the possibility that PTMs were being misidentified as mutations in our pipeline, all cell-line proteomic data were re-searched with MaxQuant [40] against the reviewed canonical UniProtKB FASTA database in “dependent-peptide” mode. Dependent peptides are assigned to MS^2^ as possible modifications to already identified peptides within a sample (i.e. modifications could be classic PTMs or amino-acid substitutions). A schematic detailing how MaxQuant dependent peptides were used to remove potentially misidentified mutants is in Additional file 1: Figure S2b, representing a conservative way of dealing with this potential issue (i.e. preference was given to the MaxQuant results and discordant peptides were removed from our results).

Potential post-translational or chemical modifications that matched to filtered variant PSMs by scan header were examined as to the position of the proposed PTM. For this analysis, dependent peptides were filtered such that the probability that the modification occurred at a specific site (the positional probability) was greater than 0.8. This relaxed threshold was used to ensure that MS^2^ spectra for proteogenomic peptides that could be assigned as chemically modified sequences from a differing starting peptide sequence or site of modification were removed. It was our observation that dependent peptides modified at the same site as proteogenomic peptides nearly always described the same mutation (i.e. the modification simply resulted in a different amino acid that was also called by the proteogenomics search). An example table showing peptides removed by our approach is shown for the exome-seq data (Additional file 10).

We also generated a list of variant peptides derived from the dependent-peptide search. These were filtered from all modifications proposed by MaxQuant as follows. First, we identified the amino-acid residue in the canonical peptide sequence that was modified. Next, we assigned single amino-acid variants based on MS^1^ mass-shift that was consistent with an amino-acid change from that starting amino acid. We used a positional probability threshold of ≥ 0.95 to stringently threshold these dependent peptides and found 1031 unique single amino-acid variants (Additional file 11).

#### Protein abundance filter

To further reduce potential false-positives, we elected to remove all proteogenomics PSMs for which there was no additional evidence of protein abundance (i.e. identification of peptides mapping to canonical sequences of the same protein). Therefore, each mutated peptide included in our final list has additional evidence of being expressed within the same cell-line.

The final list of PSMs from different search algorithms were then grouped based on the source RAW file and Scan ID and categorized into the following tiers:

- Tier 1: all peptides identified after the above filtration process.
- Tier 2: peptides identified by at least two algorithms.
- Tier 3: peptides identified by all three algorithms.
- Tier 4: peptides identified by all three algorithms with two spectra or more.

Detailed information of search output results and filtration steps for all NCI60 cell-lines is available in Additional files 4 and 5.

## Results

### Characterizing reference and variant protein sequence databases

**Fig. 1:**
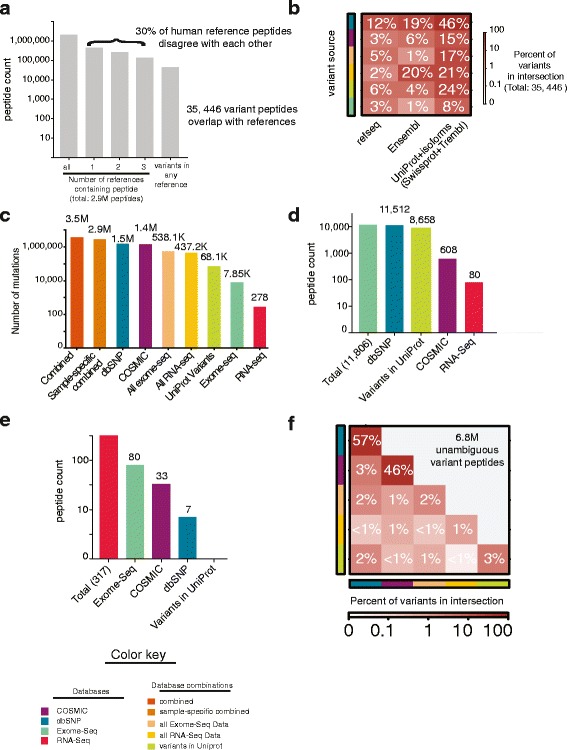
The detectable tryptic space of reference and variant human proteins. **a** Distribution of 2.9 million reference proteome tryptic peptides (length 6–35 amino-acids; including two possible trypsin missed cleavages) derived from four commonly used reference proteomes. Counts are represented using a log_10_ scale. Panels (**b**–**f**) use the prostate cancer cell-line PC-3 as an example. **b** Distribution of the 35,445 variant peptides that are also contained within at least one reference proteome. *Y-axis* covariate depicts the source of the variant. Color gradient indicates the percentage of the 35,446 variants that overlap with each reference using a log_10_ scale. **c** Numbers of protein variants in the nine major database variants used to search PC-3 proteomics data. Counts are in a log_10_ scale. **d** Total number of exome-seq derived variant peptides and their membership in other databases. Counts are in a log_10_ scale. **e** Total number of RNA-seq derived variant peptides and their membership in other databases. Counts are in a log_10_ scale. **f** Total number of peptides derived from various community-based databases and their redundancy with each other. Counts are in a log_10_ scale

Our aim was to describe protein sequence variation beyond what is already included in reference proteomes. We therefore began by examining the background of the reference human proteomes, with the aim to understand the differences between them in tryptic peptide space. We examined four commonly used reference proteomes: (1) a database of 20,187 canonical protein sequences from UniProt (Swiss-Prot); (2) a second UniProt database with 88,717 proteins including isoforms (Swiss-Prot + Trembl); (3) the reference proteome derived from the Ensembl genome model using GRCh37 (v75) with 104,763 protein sequences (henceforth denoted Ensembl); and (4) the reference proteome derived from the RefSeq annotation model (release 68) consisting of 72,128 proteins. We in silico digested each of these reference human proteomes to produce a total of 2.95 million distinct tryptic peptides within the range of 6–35 amino acids in length; peptides that are most commonly detected by MS (Fig. 1a; Additional file 1: Figure S3). Of these, 70% (2,064,452) showed 100% sequence identity between all reference proteomes. The remaining 30% (887,991) of tryptic peptides constituted a large number of potentially detectable tryptic peptides missing in at least one reference proteome.

Given this disagreement between reference proteomes at the peptide level, we recommend that variant peptides eventually reported by proteogenomics should be filtered against the Ensembl, RefSeq, and UniProt derived proteomes. To illustrate why this is necessary, after filtering against the smallest human reference proteome “canonical protein sequences” from UniProt, 7.3 million distinct tryptic peptides remained within our proteogenomic databases. However, of these, 35,446 overlapped with the other three reference human proteomes (Fig. 1b), with 43% derived from Ensembl and RefSeq and 57% were present within Uniprot + isoforms (Swiss-prot + Trembl). Variants present in reference proteomes were all in community-derived databases, though 12% were also found in sample-specific exome-seq. These peptides cannot be disambiguated from the reference and should not be included in the final set of variant peptides detected. Improper filtering of putative variant peptides is a critical and often overlooked issue in their detection. When we compare our methodology to other efforts [41], we find that while we start with nearly the same peptides, we are more conservative and exclude many variant peptides from our final lists (Additional file 1: Figure S4). However, our filtration steps are conservative, aimed to rigorously reduce false-positive identifications, especially in the context of sample specific databases. If follow-up validation strategies using synthetic peptides and targeted peptide quantifications are applied, less stringent filters may be appropriate.

Taking the prostate cancer cell-line PC3 as an example, the total number of unique protein variants contained within the major database types we generated is summarized in Fig. 1c and Additional file 3. Millions of unique and distinct tryptic peptides (7.3 million) derived from our databases represent the tryptic space of proteome variation explored in this study. Each peptide was included within at least one database, but there was much redundancy between databases (Fig. 1d–f). While thousands of peptides (12,043) with sample-specific genomic evidence were included (Fig. 1d, e), the vast majority of peptides (6.84 million) were exclusive to community-based databases (Fig. 1f).

### Scope of variant peptides identified

**Fig. 2:**
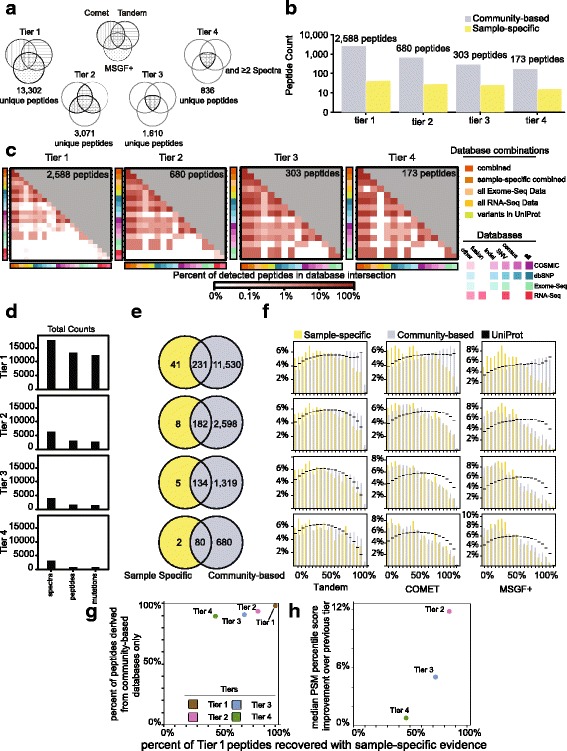
Detection of variant proteins within the nine deep proteomes. **a** Numbers of unique variant peptides identified in tiers 1–4 using MS data from the nine deep proteomes. **b** Unique variant peptides identified within the prostate cancer cell-line PC3 across tiers 1–4 (log_10_ scale). **c** *Heatmaps* depicting the percent contribution of each database towards the total number of peptides identified for that tier in PC3. The number of peptides overlapping each database pair is provided as well. Color scale is in log_10_. **d** Total number of spectra, peptides, and unique mutations identified by tier. **e** Summary of peptides identified within the nine deep proteomes within sample-specific databases or within community-based databases (tiers 1–4). **f** Percentile score distribution summary by algorithm and tier. *X-axis* ranges from high scoring peptides (0’th percentile) to lower scoring peptides (100’th percentile). A similar figure using original e-value scores is depicted in Additional file 1: Figure S6. The distribution of peptide scores from a search against a standard UniProt database is shown in *black*. **g** Increasing the stringency of identifying a peptide influences the percentage of peptides present in community-based databases between tiers 1 and 2 more than moving to subsequent tiers. **h** When compared, tier 2 peptides tend to be higher ranked by 12% than tier 1 peptides; this improvement in peptide rank drops off quickly from tier 2 to tier 3 (4%) and tier 3 to tier 4 (1%)

In total, 13,302 unique variant peptides were identified within the deep NCI60 proteomic dataset (Additional files 6 and 7). To understand how these peptides differed in terms of confidence of identification, we quantified the evidence for peptide identification using four tiers of stringency (Fig. 2a). Tier 1 peptides were assigned by the union of the three search algorithms (13,302 peptides). Tier 2 and tier 3 peptides were identified by either two (3071 peptides) or three algorithms (1610 peptides), and tier 4 peptides were identified by three algorithms and more than one PSM (836 peptides). These overall trends were also representative for one cell-line, as shown for PC3 (Fig. 2b). The peptides identified in PC3 came from a diversity of databases and would often be present in smaller database searches as well as larger ones (Fig. 2c). The overall numbers of PSMs, unique peptides, and mutations detected within the nine deep proteomes has also been summarized (Fig. 2d). We further evaluated all PSMs to check for biases in hydrophobicity, charge, and length (Additional file 1: Figure S5). We found that variant peptides identified through our pipeline, tended to be larger and of higher charge than those identified using standard proteomic searches (see “Discussion”).

We focused on community-derived databases or sample-specific database searches (Additional file 1: Figure S1b). Fewer peptides (272) were identified with genomic evidence than from the tryptic space of community-derived variants (11,761; Fig. 2e). The proportion of peptides with genomic evidence increased from tier 1 to tier 4. This mild improvement for peptides with genomic evidence came at the cost of proteogenomic peptide identification (Fig. 2d).

We evaluated how peptides with and without dataset-specific genomic evidence differed in their score distributions (Fig. 2f). We focused on those peptides that were derived from community-based databases, some of which also had genomic evidence. For each search, peptides were percentile ranked, with a percentile rank of 1% indicating a peptide in the top 1% of peptides in that search. At tier 1 there was only a slight bias showing better PSM scores if the peptide had sample-specific genomic evidence, supporting the validity of these community-based peptide identifications (Fig. 2f; Additional file 1: Figure S6). MS-GF+ consistently identified more peptides than COMET and Tandem. The fraction of peptides with population variation evidence and the fraction of peptides with genomic evidence initially identified in tier 1 decreased relatively linearly with tier (Fig. 2g). A 12% improvement in peptide median score occurred between tiers 1 and 2 (Fig. 2f/h). This compared to a ~6% improvement from tier 2 to tier 3, indicating the benefit of incorporating additional algorithms rapidly depleted. Similar trends for these score distributions were observed for a standard UniProt search (Fig. 2f; Additional file 1: Figure S6). We recommend using tier 2 as a balance between sensitivity and specificity, although we suggest that all proteogenomics PSMs should be closely examined (possibly using synthetic peptides) before subsequent analysis.

### The relevance of proteogenomic peptides

**Fig. 3:**
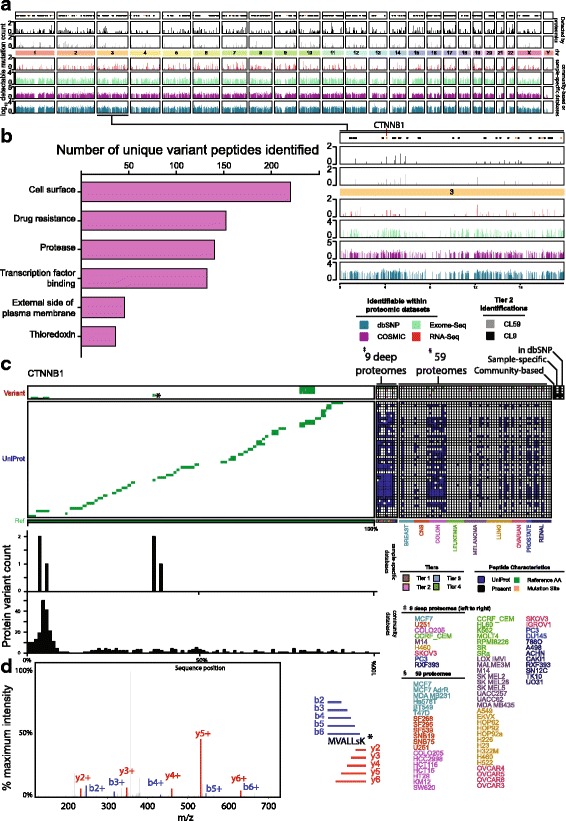
Identification of cancer-related variant peptides. **a** Genome coverage of potentially detectable proteogenomic peptides (6–35 amino acids) within the generated search databases (bottom). Variant proteins identified at tier 2 within 59 shallow and nine deep proteomes have been summarized in *black* and *gray*, respectively (*top*). *Black dots* correspond to the locations of COSMIC cancer census genes and *orange dots* indicate those detected at tier 2. **b** Variants identified were assessed by the drug gene interaction [43] database to identify variants that might potentially be targetable or affect related pathways. Counts relate to the number of variant peptides identified in each category for tier 2 peptides. Only categories significantly enriched at *p* < 0.01 are depicted. **c** Variant peptides detected for CTTNB1. Mutation locations have been depicted in *orange*. Identification of reference peptides for the same protein are shown in *blue*, with an alignment describing the peptides detected. *Bar plots* illustrate the variants that were present in genomics for this gene (*top*) and all mutations present in community-based databases (*bottom*). **d** A tier 2 peptide identified for CTTNB1 showing clear coverage of *y* and *b* ions

Any proteogenomic pipeline must detect peptides in an unbiased manner across the entire genome as well as variations in relevant cancer genes and pathways. Peptide variants identified within the NCI60 dataset were broadly distributed across the genome (Fig. 3a), but clearly the detected variants are just a fraction of those theoretically detectable within the datasets searched. In total, we found 4771 unique protein variations mapping to 2200 genes at tier 2 (Additional file 12) across both the deep (1511 HGNC gene ids) and the shallow (1469 HGNC gene ids) proteomes. The median number of mutations per gene was just 1 in both proteome datasets. However, there were a few genes where an excess of variants was identified across cell-lines. *AHNAK*, a large 700-kDa structural scaffold nucleoprotein with known roles in cell-migration and metastasis topped the list with 91 variants identified across the nine deep proteomes. In total, 211 COSMIC cancer gene census genes harbored detected variants, demonstrating the potential of proteogenomics for variant detection in cancer. These genes tended to be highly expressed within the nine deep proteomes, as estimated using iBAQ scores from a standard UniProt search (Additional file 1: Figure S7).

Variants identified were assessed by the drug gene interaction database [42, 43] in order to identify those variants that could be targetable by a drug or affect targetable pathways. We tested whether the genes associated with variant peptides identified at tier 2 (3071 unique peptides) were enriched in specific druggable gene categories when compared to equally sized random subsamples of unique peptides identified in a standard UniProt search against the nine deep proteomes. As a null distribution, we took 100,000 subsamples of 3071 peptides from a UniProt search and binned them into categories within the drug gene interaction database. Using this methodology, several druggable gene categories were statistically enriched (*p* < 0.01) in variant peptide detections at tier 2 (Fig. 3b). Statistically enriched categories included variants from various tumor suppressors, cell-surface proteins, proteins involved in drug resistance, and proteins involved in transcription factor binding.

We mapped variant peptides back onto the canonical reference sequence for the oncogene beta-catenin (CTNNB1) (Fig. 3c), revealing several mutations in both the deep and shallow proteomes in cell-lines derived from different cancers. While many variants were identified, they were only a small fraction of the possible variants for CTNNB1 (Fig. 3c, bar plots). As an example, we refer to a tier 2 PSM with both exome-seq and RNA-seq evidence for which we have identified a peptide sequence (Fig. 3d).

**Fig. 4:**
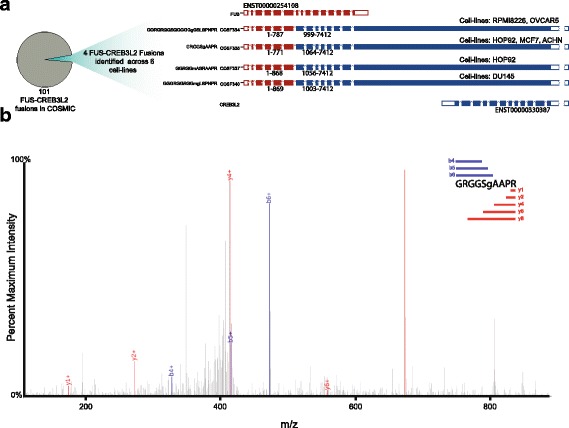
Identification of fusion peptides. We identified several fusions of FUS to CREB3L2 of which there are 101 reported in the COSMIC database. **a** Of these 101 fusions, four were repeatedly identified across six cell-lines. **b** MS^2^ spectrum for one fusion peptide is displayed

We identified 111 fusion proteins in the nine deep proteomes and 508 fusion proteins in the 59 shallow proteomes (Additional files 8 and 9). The gene encoding the RNA-binding protein FUS is located at a common site of chromosomal translocations in human low grade fibromyxoid sarcomas and frequently forms chimeric fusions with one of several different genes [44]. We identified four different FUS-CREB3L2 fusions across seven cell-lines, from a total of 101 FUS-CREB3L2 fusions present in COSMIC (Fig. 4a/b; Additional file 1: Figure S8). These fusions were identified independently of RNA-seq, for which fusion calls from sample-specific transcriptomics (median three per cell-line) were rare [37]. Based on our sample-specific RNA-seq searches, only three fusions were identified across the nine deep proteomes and 33 across the 59 shallow proteomes.

## Discussion

Proteogenomic approaches promise the personalized detection of genomic aberrations within protein samples and may represent an important untapped area in cancer biomarker discovery. We explored the limits of variant peptide detection using MS-based proteogenomics strategies. In general, there are three interrelated aspects of PSM assignment at play: (1) the capacity to separate peptides in chromatography and mass-to-charge space; (2) the sensitivity of the mass-spectrometer itself; and (3) the overall sequence coverage of the tryptic peptidome. Proteomics search-algorithms must identify the amino-acid sequence with the highest likelihood to have produced a particular MS^2^ spectrum, carefully taking these challenges into account. Algorithms must screen protein sequence databases and identify a set of putative peptides of the same mass (within error) of the peak in the MS^1^ spectrum associated with the MS^2^ in question. In variant peptide identification, as database size increases, the algorithm must choose from an increasingly large pool of potential peptides, which must be assigned to spectra that often may originate from more than one peptide molecule.

Interestingly, variant peptides identified through our pipeline tended to be larger and of higher charge than those identified using standard proteomic searches. While the exact reason for this observation is currently not known, we speculate that for larger databases a better search score is required to pass a predefined 1% FDR (based on a target-decoy approach). Larger peptides, which in general are associated with a higher score could hence be favored in this process. However, as a caveat, longer peptides tend to have slightly lower overall *y* and *b* ion coverage, which could also lead to potential false-positives.

We have developed a series of recommendations to serve as guidelines to better characterize variant proteoforms within cancer proteomics datasets using custom sequence databases and a target-decoy approach. (1) We recommend variant peptides be identified using more than one search algorithm using a split target-decoy approach [15]. (2) We further recommend the use of several filters to reduce sources of possible false-positive identification not accounted for by commonly used proteomics approaches. This includes filters that remove variant peptides detected within standard reference proteomes or that could be accounted for by a PTM of a given peptide sequence. (3) We also recommend that identified protein variants be supported with additional evidence for the expression of their source protein.

Ultimately, generation of custom protein sequence databases and filtering of resulting data to balance the sensitivity and specificity of peptide detection will depend on the investigator and goal of the project. For example, it may be appropriate when using databases with sample-specific genomic evidence to keep peptides that match to reference proteomes for further investigation. Conversely, in the absence of sample specific data, variant peptides could be identified using large publicly available databases, although with a higher risk of false-positive identifications. As a final recommendation, we suggest that promising candidates be visually inspected and preferentially compared to spectra generated by synthetic peptides. This will provide additional validation and the possibility for the development of targeted proteomics assays.

Our study illustrates the need for further improvements in proteogenomics pipelines. With our stringent search criteria, we identified 4771 protein variants corresponding to somatic and germline deviations from reference proteomes in 2200 genes among the NCI60 cell-line proteomes. This is despite the tens of thousands of identifiable peptide variants with sample-specific genomic evidence present in our search databases. The detection of protein variants is particularly difficult as each can only be detected by six unique tryptic peptides after accounting for up to two missed cleavages. Proteins may be lost during protein extraction and peptide biases may be introduced during digestion, detection, and PSM assignment. These technical challenges, as others have noted [32], lead to a lack of sequence coverage among all proteins identified and result in a lack of sensitivity for variant peptide identification. Compounding on a lack of sensitivity is the potential for false identification. As has been shown for PTMs, it is plausible that the use of alternative proteases could increase the likelihood of detecting specific mutations by shotgun proteomics [45]. There are other strategies for detecting variants from MS datasets. The proteogenomic approach can easily be integrated with semi-supervised methods that search for variants of reference proteins present in standard search databases. The dependent peptide searches we used to filter out potential PTMs allow for a comparison to these approaches. We collected 1031 high confidence single-amino-acid-variant dependent peptides (positional probability > 0.95) (Additional file 12). In Total, 97 variant peptides or 10.3% of dependent peptide variants overlapped with proteogenomic variants, highlighting the potential for these methodologies to expand our capacity for variant protein detection. Other semi-supervised or “open search algorithms,” such as the recently released MSFragger [46] and spectral network inference [47], could also be used as additional strategies for the parallel identification of PTMs or proteoform variants. While beyond the scope of the current manuscript, head-to-head comparisons of open search algorithms, custom database proteogenomics searches, and spectral libraries using massive synthetic peptide libraries [48] are now possible and will likely lead to the refinement of current proteogenomic strategies.

## Conclusions

Proteogenomics can identify germline and somatic mutations within important cancer genes (Fig. 3). While the underlying technology improves, the proteogenomics community can now focus on integrating alternative strategies for detecting protein variants. The proteogenomic approach described here can be integrated with semi-supervised methods that search for variants of canonical proteins and de novo sequencing (i.e. PEAKS [49]) based methodologies that could identify variants missed by genomics. Added sensitivity could be achieved by constructing spectral libraries from synthetic peptides derived from genomic evidence, which could help with the development of more statistically refined proteogenomics pipelines.

## Additional files

Additional file 1: Supplementary figures. **Figure S1.** Generation of proteogenomic databases. **Figure S2.** Proteogenomic search and filtering strategy. **Figure S3.** Comparison of reference proteomes. **Figure S4.** Comparison to other studies. **Figure S5.** Biophysical properties of detected variant peptides. **Figure S6.** Score distributions across community-based database searches. **Figure S7.** Variants identified for genes in the COSMIC cancer gene census tend to be highly expressed in the same cell-line. **Figure S8.** MS^2^ spectra for FUS-CREB3L2 fusions. (PDF 8243 kb)

Additional file 2: Summary of databases generated and searched in the study. (DOCX 25 kb)

Additional file 3: Number of coding sequence variants in each database generated. (XLSX 25 kb)

Additional file 4: Number of peptides identified after each filtering step of post-processing summarized for the nine deep proteomes. (XLS 59 kb)

Additional file 5: Number of peptides identified after each filtering step of post-processing summarized for the 59 shallow proteomes. (XLS 205 kb)

Additional file 6: Variant peptides detected in searches against the nine deep proteomes. (XLSX 5224 kb)

Additional file 7: Variant peptides detected in searches against the 59 shallow proteomes. (XLSX 13008 kb)

Additional file 8: Fusions detected across the nine deep proteomes. (XLSX 25 kb)

Additional file 9: Fusions detected across the 59 shallow proteomes. (XLSX 78 kb)

Additional file 10: Example output from PTM filter. (XLSX 82 kb)

Additional file 11: Variant peptide detections from MaxQuant. (XLSX 159 kb)

Additional file 12: Numbers of tier 2 variants detected per gene for both proteomic datasets studied. (XLSX 87 kb)

## Abbreviations

COSMIC: Catalogue Of Somatic Mutations In Cancer
CTNNB1: Beta-catenin
FDR: False discovery rate
MS: Mass spectrometry
PSMs: Peptide spectrum matches
PTM: Post translational modification
