# Supplementary material for: Detecting protein variants by mass spectrometry: a comprehensive study in cancer cell-lines
Source: Genome Med. 2017 Jul 18;9:62. doi: 10.1186/s13073-017-0454-9 (PMC5514513; doi:10.1186/s13073-017-0454-9)
Supplement: Supplementary file 1 — Supplementary figures. Figure S1. Generation of proteogenomic databases. Figure S2. Proteogenomic search and filtering strategy. Figure S3. Comparison of reference proteomes. Figure S4. Comparison to other studies. Figure S5. Biophysical properties of detected variant peptides. Figure S6. Score distributions across community-based database searches. Figure S7. Variants identified for genes in the COSMIC cancer gene census tend to be highly expressed in the same cell-line. Figure S8. MS2 spectra for FUS-CREB3L2 fusions. (PDF 8243 kb) [file 13073_2017_454_MOESM1_ESM.pdf]

Figure S1

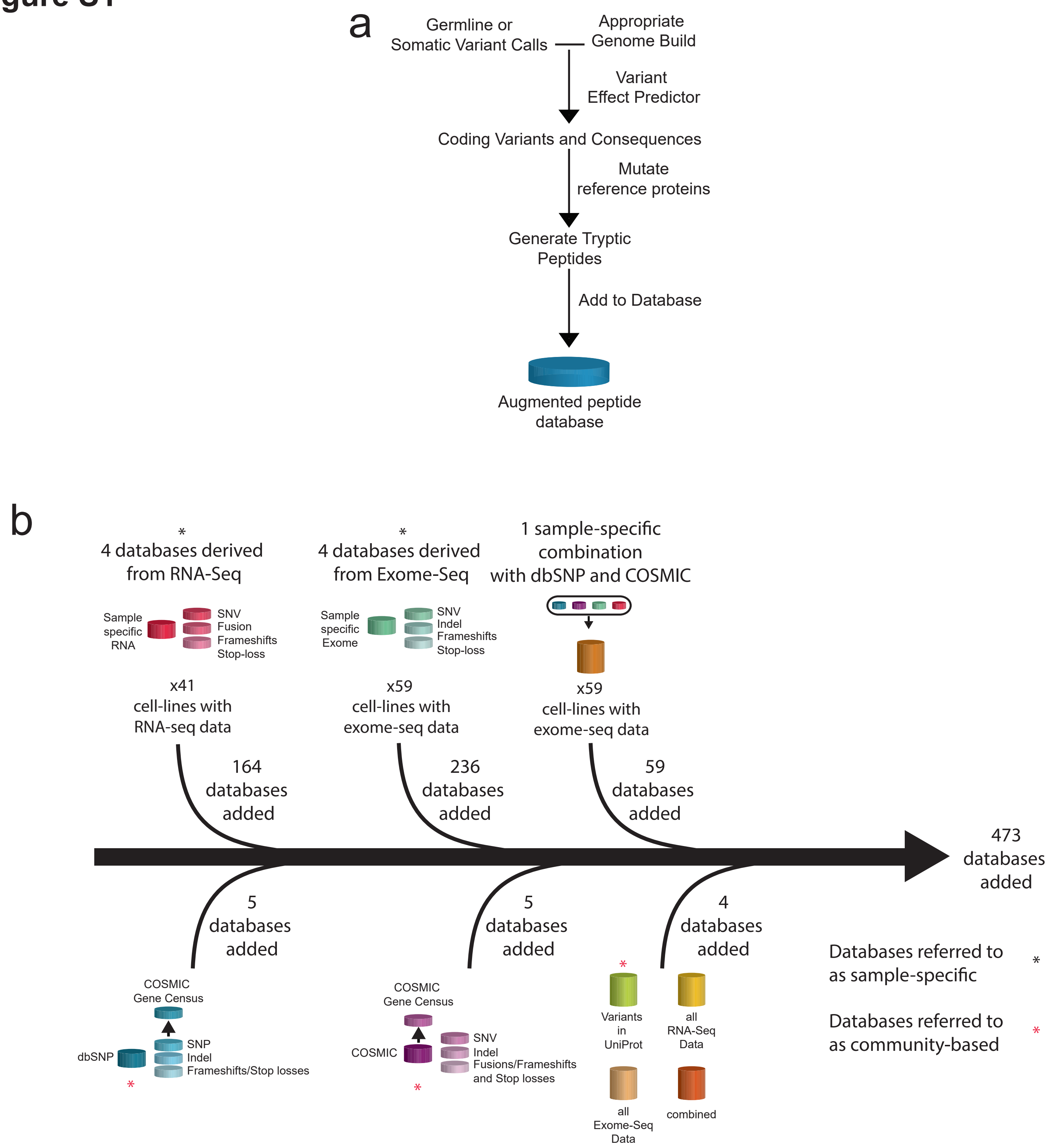

Figure S1: Generation of proteogenomic databases
(a) Scheme for the generation of databases suitable for MS-based detection of protein variants.
(b) Overview of databases generated.

Figure S2

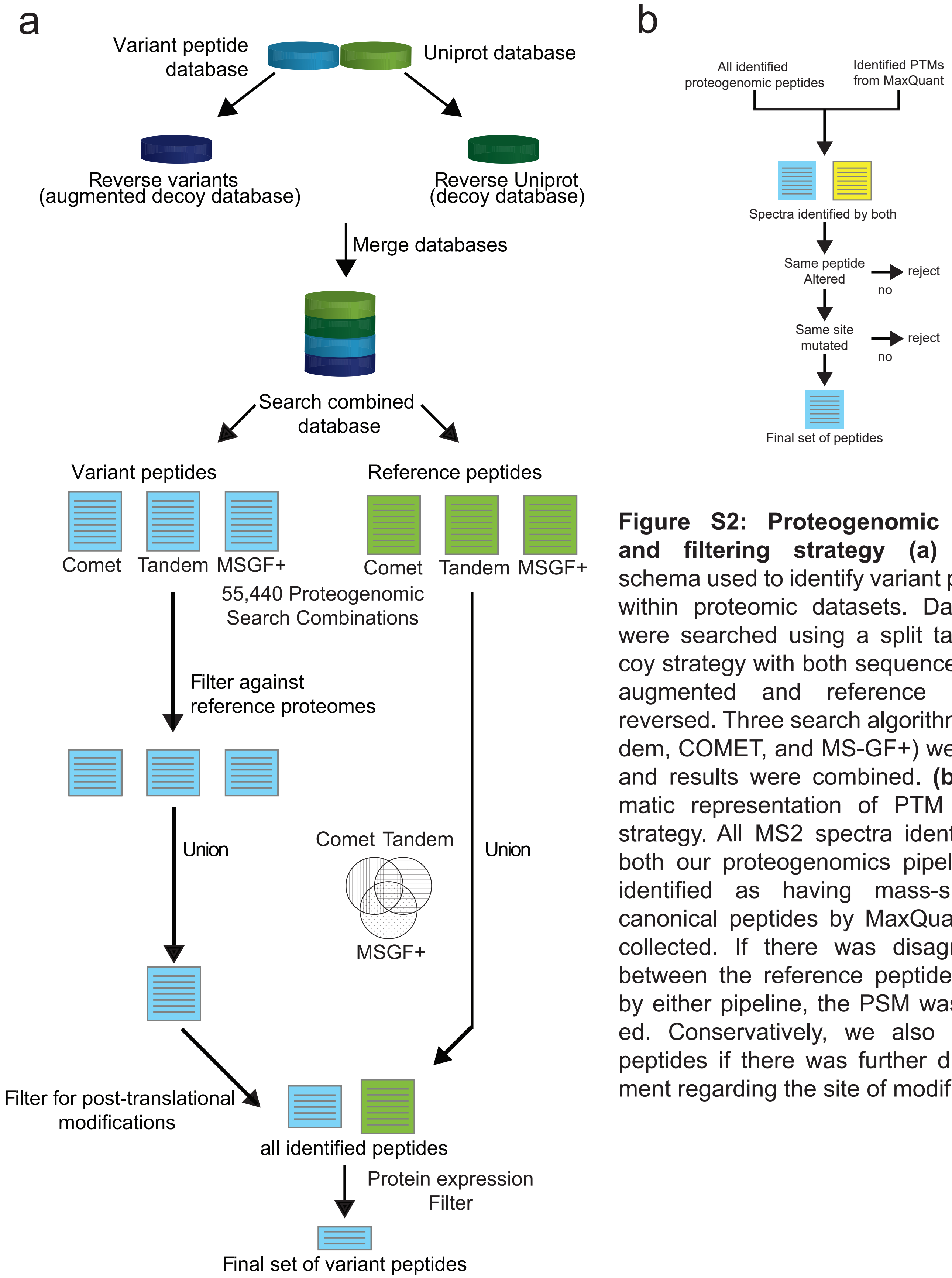

Figure S2: Proteogenomic search and filtering strategy (a) Search schema used to identify variant peptides within proteomic datasets. Databases were searched using a split target-decoy strategy with both sequences to the augmented and reference proteins reversed. Three search algorithms (Tandem, COMET, and MS-GF+) were used and results were combined. **(b)** Schematic representation of PTM filtering strategy. All MS2 spectra identified by both our proteogenomics pipeline and identified as having mass-shifts to canonical peptides by MaxQuant were collected. If there was disagreement between the reference peptide altered by either pipeline, the PSM was rejected. Conservatively, we also rejected peptides if there was further disagreement regarding the site of modification.

Figure S3

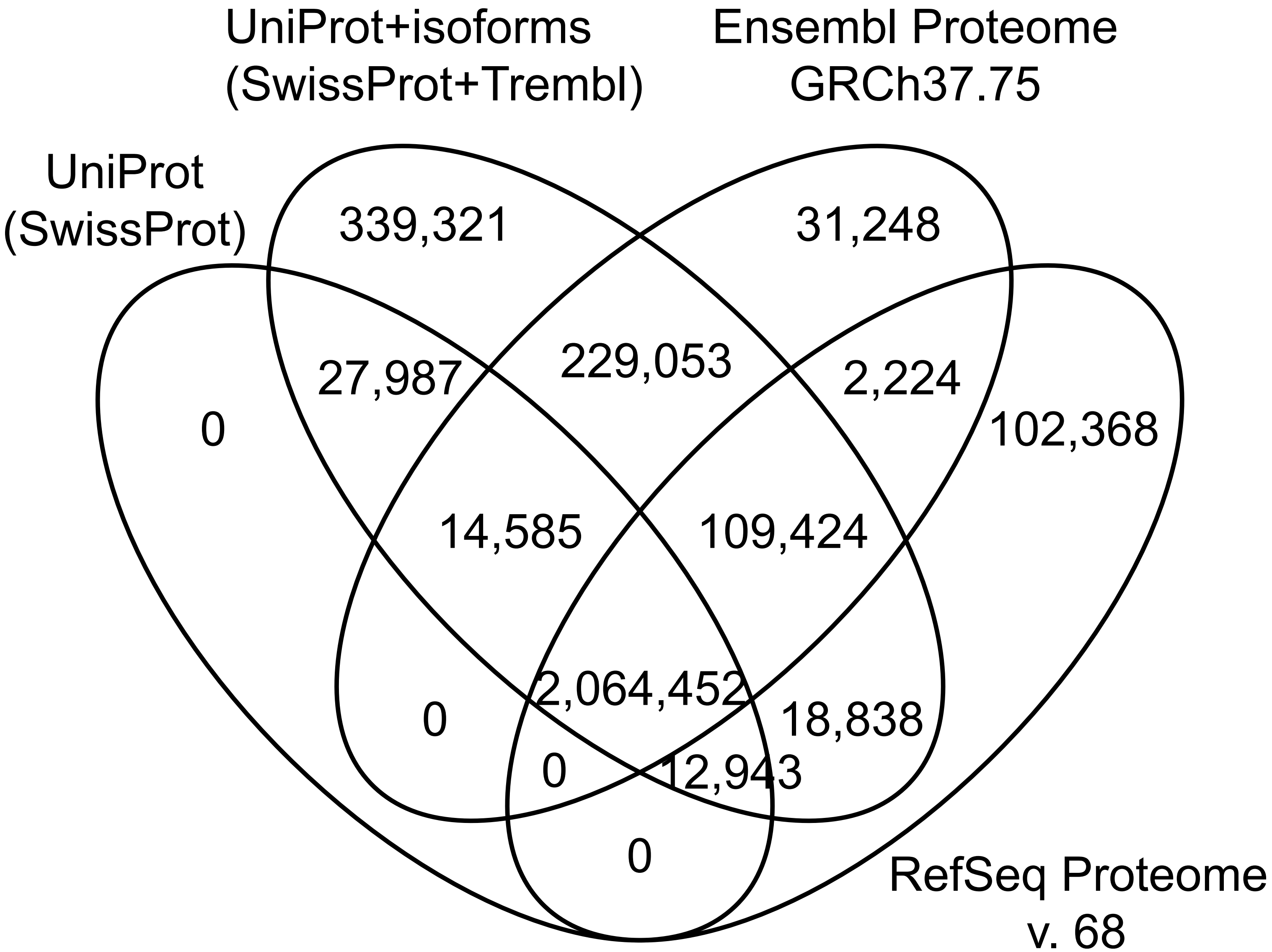

Figure S3: Comparison of reference proteomes. Venn diagram comparing the four reference databases used in this study. Proteins in each reference proteome were in silico digested and filtered in the length range 6-35 amino-acids. The Venn diagram portrays the overlaps of unique peptides originating from each reference database.

Figure S4

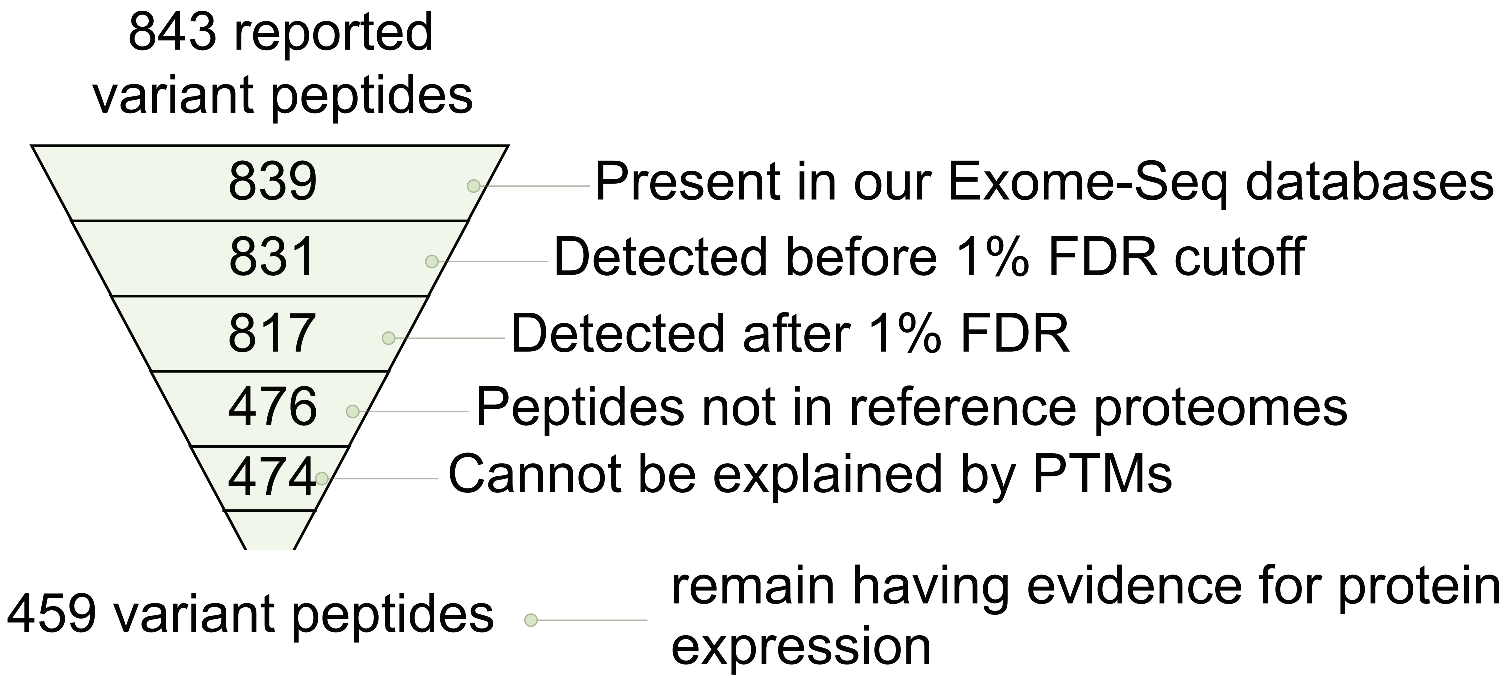

Figure S4: Comparison to other studies. To illustrate the importance of peptide filters, we compared our results to a previous study [41]. In their study, both a cell-line specific database search and a database combining all exome sequencing data were used. The figure reports the number of peptides identified by [41] that remain after the various filtration approaches in our pipeline.

Figure S5

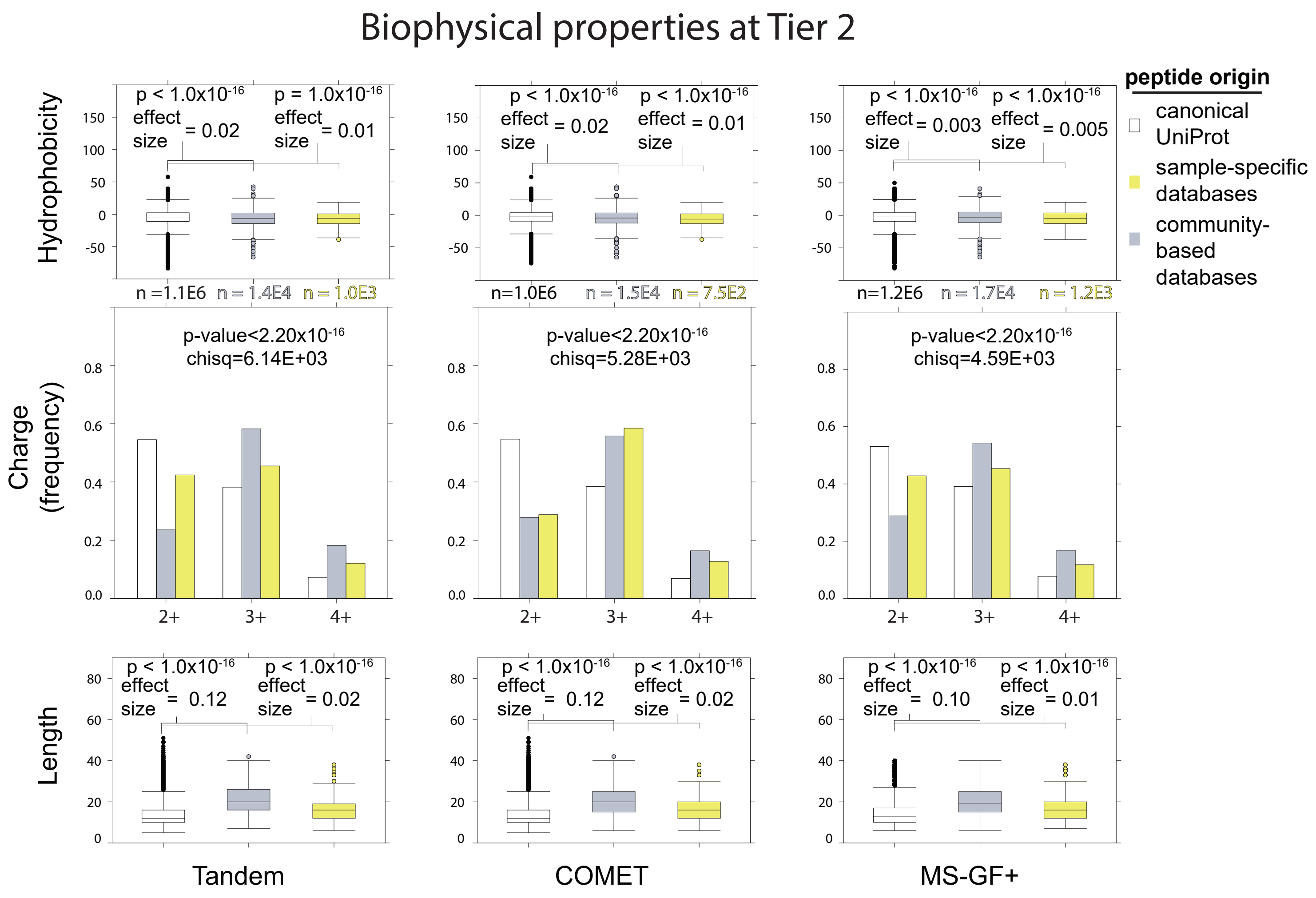

Figure S5: Biophysical properties of detected variant peptides. Top row: Boxplots show the calculated Kyte-doolittle hydrophobicity index of peptides and peptide variants identified by a PSM in the NCI60 'deep proteome' dataset (p-value from Wilcoxon sum-rank test). Middle row: Barplots show the charge of peptides and peptide variants identified by a PSM in the NCI60 'deep proteome' dataset. Variant peptides were observed to contain more charge +3 peptides than non-variant peptides, which contained more charge +2 peptides. Bottom row: Boxplots show the length of peptides and peptide variants identified by a PSM in the NCI60 'deep-proteome' dataset (p-value from chi-square test). Variant peptides were observed to contain longer peptides when compared to those peptides identified in a standard search against canonical UniProt.

Figure S6

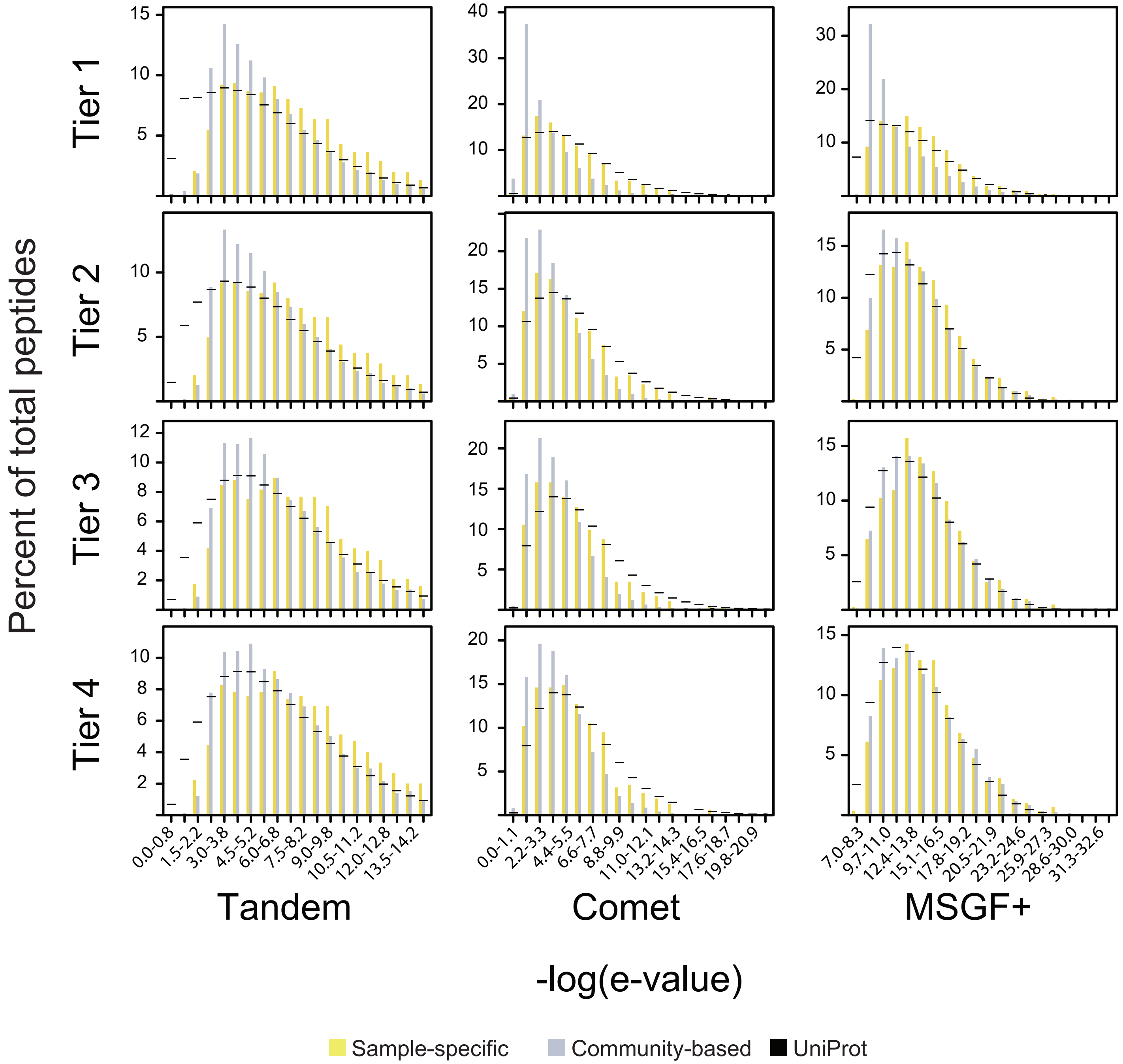

Figure S6: Score distributions across community-based database searches. E-value score distribution summary by algorithm and tier. X-axis ranges from low scoring peptides to higher scoring peptides. Y-axis are the number of unique peptides in that bin. Colors represent peptides unique to community based databases (gray) or shared with sample-specific databases (yellow). Black lines represent the results from a search against a standard UniProt protein sequence database, using the indicated search algorithm and filtration tier.

Figure S7

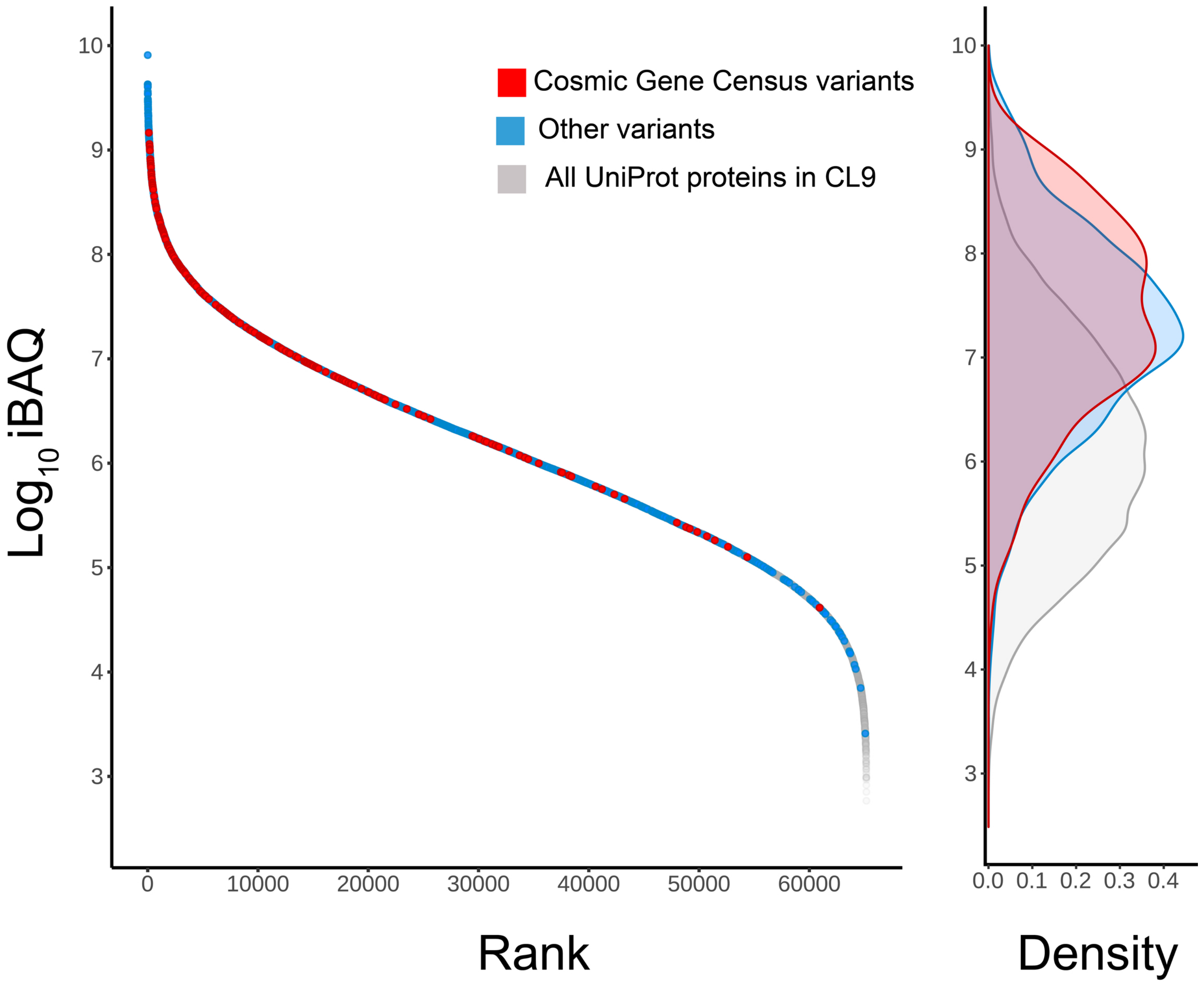

Figure S7: Variants identified for genes in the COSMIC cancer gene census tend to be highly expressed in the same cell-line. Protein abundances for the 9-deep proteomes (log10 iBAQ) were calculated from a MaxQuant search against a standard UniProt database and ranked from most abundant to least abundant. Proteins expressed in each cell line for which no variant was detected have been colored in gray. Variant peptides identified but not in the COSMIC cancer gene census are colored in blue. Variant peptides identified in the cancer gene census are colored in red. Density plots are diagrammed to the right of the plot.

Figure S8

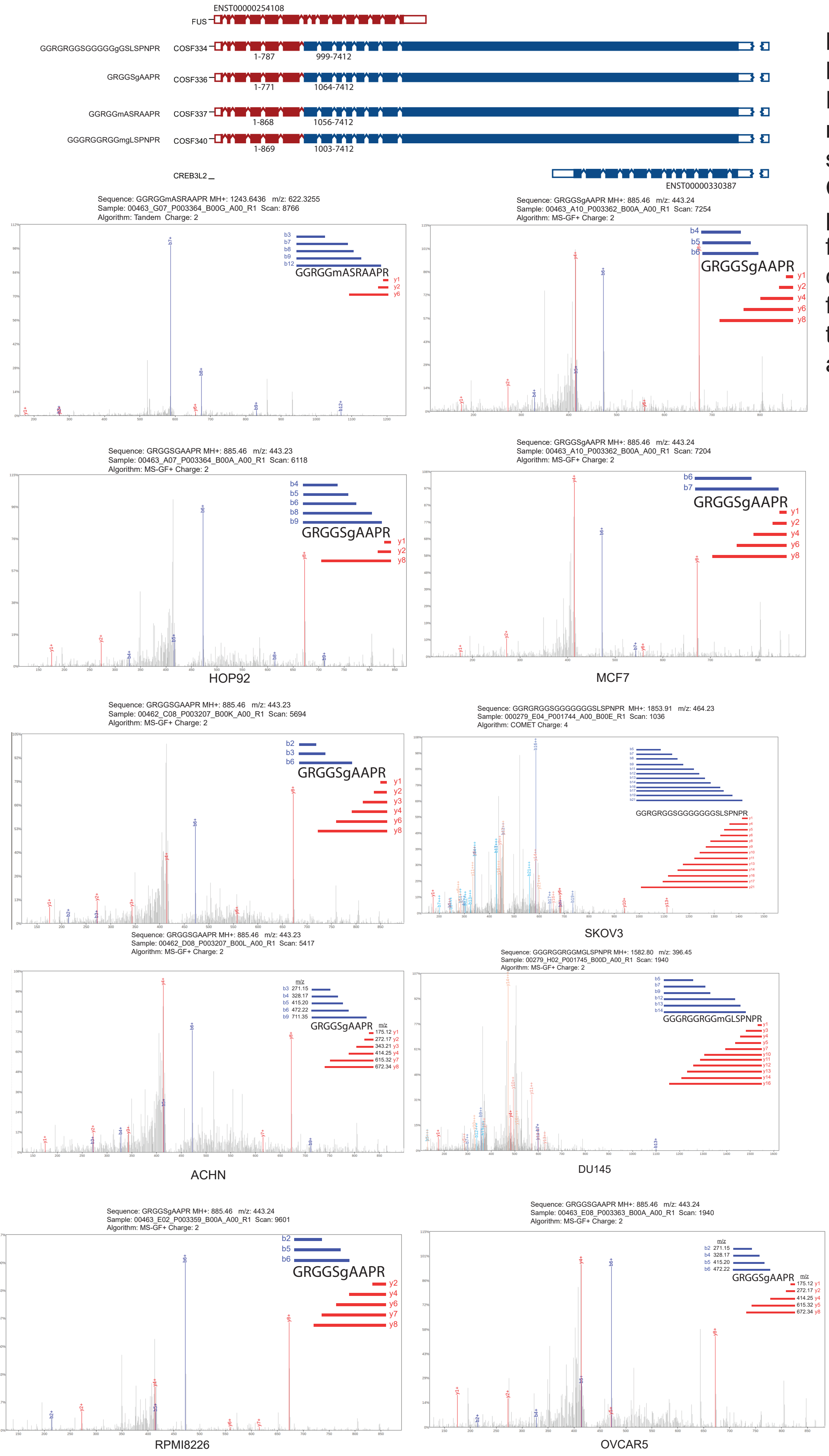

Figure S8: MS² spectra for FUS-CREB3L2 fusions. FUS-CREB3L2 fusions were repeatedly identified from searches including the COSMIC database. Here we present schematics of the fusions identified with lower case letters at the 5' end of the fusion site. In each case, peptide fragments are identified across the fusion.
