# Supplementary material for: Detecting protein variants by mass spectrometry: a comprehensive study in cancer cell-lines
Source: Genome Med. 2017 Jul 18;9:62. doi: 10.1186/s13073-017-0454-9 (PMC5514513; doi:10.1186/s13073-017-0454-9)
Supplement: Supplementary file 2 — Summary of databases generated and searched in the study. (DOCX 25 kb) [file 13073_2017_454_MOESM2_ESM.docx]

## **Supplementary File 1**

## **Fasta files generated for this study**

## *Community-based databases*

Community-based databases contain sequences corresponding to mutations cataloging somatic and germline variations in the genome found within COSMIC or dbSNP.

### *COSMIC*

**all:** Peptides harbouring mutations generated from COSMIC including SNVs, indels, fusions, frame-shift causing mutations and stop-losses.

**snv:** SNVs only.

**indel:** Insertions and deletions (indels) only.

**other:** Fusions, frame-shift causing and stop-losses only.

**census:** Subset of mutations in all affecting genes present in the cosmic cancer gene census (v70).

### *dbSNP*

**all:** Peptides harbouring mutations generated from dbSNP including SNVs, indels, frame-shift causing mutations and stop-losses.

**snv:** SNVs only.

**indel:** indels only

**other:** Frame-shift causing mutations and stop-losses only.

**census:** The collection of mutations in dbSNP within genes present in the COSMIC cancer gene census (v70).

### *Uniprot Variants*

**all:** Variants extracted from Uniprot.

## *Cell-line specific databases*

Cell-line specific databases contain sequences corresponding to somatic or germline mutations detected from genomics for that particular cell line. Mutations were generated using either exome-seq or RNA-seq. Each dataset differs in the mutation types available. While both datasets contain somatic mutations as SNVs and stop-losses, exome-seq additionally contains indels, frameshift causing mutations and stop-losses for both. The RNA-seq database additionally contains fusions but is lacking germline mutations, which are present in the exome-seq dataset.

### *Exome Set*

**all:** Germline or somatic SNVs, indels, frameshift-causing mutations and stop-losses.

**snv:** Germline or somatic SNVs.

**Indel:** Germline or somatic indels.

**other:** Germline or somatic frameshift-causing mutations and stop-losses.

### *RNA set*

**all:** Somatic SNVs, fusions and stop-losses.

**snv:** Somatic SNVs only.

**fusion:** Fusions only.

**other:** Stop-losses only.

## *Larger Combined Sets*

**Combined:** The union of exome set + RNA set + COSMIC + dbSNP for all cell-lines.

**Combined personal:** The concatenation of exome set + RNA set + COSMIC + dbSNP for cell-lines generated in a database specific way.

**Exome general:** Combination of all Exome data [1].

**RNA general:** Combination of all RNA-seq data for available NCI-60 cell-lines as well as many other cell-lines (675 total) from [2].

**References:**

1. Abaan OD, Polley EC, Davis SR, Zhu YJ, Bilke S, Walker RL, Pineda M, Gindin Y, Jiang Y, Reinhold WC, et al: **The exomes of the NCI-60 panel: a genomic resource for cancer biology and systems pharmacology.** *Cancer Res* 2013, **73:**4372-4382.

2. Klijn C, Durinck S, Stawiski EW, Haverty PM, Jiang Z, Liu H, Degenhardt J, Mayba O, Gnad F, Liu J, et al: **A comprehensive transcriptional portrait of human cancer cell lines.** *Nat Biotechnol* 2015, **33:**306-312.
